# Synergistic Effect of Cold Atmospheric Plasma and Drug Loaded Core-shell Nanoparticles on Inhibiting Breast Cancer Cell Growth

**DOI:** 10.1038/srep21974

**Published:** 2016-02-26

**Authors:** Wei Zhu, Se-Jun Lee, Nathan J. Castro, Dayun Yan, Michael Keidar, Lijie Grace Zhang

**Affiliations:** 1 Department of Mechanical and Aerospace Engineering, The George Washington University, Washington DC 20052, USA; 2 Department of Biomedical Engineering, The George Washington University, Washington DC 20052, USA; 3 Department of Medicine, The George Washington University, Washington DC 20052, USA

## Abstract

Nano-based drug delivery devices allowing for effective and sustained targeted delivery of therapeutic agents to solid tumors have revolutionized cancer treatment. As an emerging biomedical technique, cold atmospheric plasma (CAP), an ionized non-thermal gas mixture composed of various reactive oxygen species, reactive nitrogen species, and UV photons, shows great potential for cancer treatment. Here we seek to develop a new dual cancer therapeutic method by integrating promising CAP and novel drug loaded core-shell nanoparticles and evaluate its underlying mechanism for targeted breast cancer treatment. For this purpose, core-shell nanoparticles were synthesized via co-axial electrospraying. Biocompatible poly (lactic-co-glycolic acid) was selected as the polymer shell to encapsulate anti-cancer therapeutics. Results demonstrated uniform size distribution and high drug encapsulation efficacy of the electrosprayed nanoparticles. Cell studies demonstrated the effectiveness of drug loaded nanoparticles and CAP for synergistic inhibition of breast cancer cell growth when compared to each treatment separately. Importantly, we found CAP induced down-regulation of metastasis related gene expression (VEGF, MTDH, MMP9, and MMP2) as well as facilitated drug loaded nanoparticle uptake which may aid in minimizing drug resistance-a major problem in chemotherapy. Thus, the integration of CAP and drug encapsulated nanoparticles provides a promising tool for the development of a new cancer treatment strategy.

Currently breast cancer remains one of the leading diseases affecting women worldwide. Chemotherapy and radiotherapy are common approaches for the treatment of early stage breast cancer leading to improved disease-free and overall survival^1^. Fluorouracil (5-FU), a pharmaceutical that has been widely used in breast, gastrointestinal, gynecological as well as head and neck cancers, belongs to the family of chemotherapeutics known as DNA synthesis inhibitors which halt cell growth^2^. Especially, 5-FU has been used for breast cancer remediation over 40 years^3^. Like most chemotherapeutics, 5-FU treatment has led to a high incidence of severe toxic effects to gastrointestinal, neural, hematological, cardiac, and dermatological systems through direct intravenous administration^4^. Therefore, sustained and targeted drug delivery systems function to reduce systematic side effects and improve treatment efficiency.

Polymeric nanoparticles have been extensively used in medicine as drug delivery devices^5^. They have the potential of improving hydrophobic drug delivery, reducing metabolic drug degradation, targeting specific cells by chemical modification, and exhibiting sustained and triggered release^6^. Therefore, delivery of anti-cancer agents using nanoparticle carriers has been extensively investigated. Although still a burgeoning field, some chemotherapeutic containing nanoparticles are currently undergoing clinical trials or have been approved Food and Drug Administration (FDA) for breast cancer treatment^7^,^8^. In lieu of intravenous administration, nanoparticle drug delivery devices are able to manipulate the pharmacokinetic behavior of encapsulated drugs which can address the negative side effects of systemic delivery^9^,^10^. Multiple techniques including top-down (lithography, etching, milling or machining, electrospraying) and bottom-up (gas/vapor phase fabrication-pyrolysis, liquid phase fabrication, Sol-Gel or solvothermal synthesis) processes have been investigated to synthesize polymeric nanoparticles. Amongst them, electrospraying is one of the most popular and efficient techniques for nanoparticle fabrication. In the presence of high voltage, polymeric solutions can readily form nanoparticles with high drug encapsulation efficacy.

In addition to traditional chemotherapy and radiotherapy, cold atmospheric plasma (CAP) is an emerging biomedical technique for selective cancer treatment^11^. CAP is a plume-like cocktail containing reactive oxygen species, reactive nitrogen species, charge particles, UV, etc.^12^. The unique non-equilibrium, non-thermal feature of CAP is of great interest in biomedical application. Unlike thermal plasmas which utilize heat to ablate and cauterize tissues, CAP has a temperature close to room temperature rendering it capable of selective tissue treatment. Therefore, CAP has been used in wound healing, inert surface sterilization, and tissue regeneration^13^,^14^. Our recent studies have illustrated the great promise of CAP in cancer remediation^15^,^16^. The aim of CAP involvement for cancer therapy is to induce chemically specific cellular responses for selective cancer killing and minimal healthy tissue damage. The effectiveness of CAP in these biomedical applications is most likely attributable to its complex composition, in which neutral atoms and molecules including singlet oxygen (^1^O_2_), hydroxyl radicals (OH.), nitric oxide (NO) can interact directly with cells and tissues associating with the influx of various electrons, positive and negative ions^17^,^18^,^19^. Although the underlying mechanism of CAP-cell interaction is still not totally understood, both *in vivo* and *in vitro* studies from our labs have revealed CAP can selectively ablate cancer cells and significantly reduce solid tumor size with a minimal damage to normal cells^15^,^16^. Some studies have demonstrated that CAP leads to cancer cell death through apoptosis^16^. Reactive oxygen species are postulated to play a major role during in the cellular response to CAP. Noticeable accumulation of intracellular reactive oxygen species upon the CAP treatment has been widely observed^12^. Experimental evidence suggests reactive oxygen species and reactive nitrogen species operating as biologically active agents can reduce solid tumor size by mediating oxidative and nitrosative stress around neoplastic tissue^20^. Therefore, like conventional therapeutics, the anti-cancer effects of CAP treatment are thought to be attributed to pro-oxidant, oxidative, and nitrosative stress mechanisms^20^. Furthermore, there is documented evidence suggesting CAP is capable of preventing the development of treatment resistant cells, a major problem of current therapies^20^,^21^.

Recently, the synergistic combination of nanoparticle chemotherapeutic delivery systems and plasma technology has presented its potential in medicine, particularly in cancer therapy^22^. Kim *et al.* showed that non-thermal plasmas coupled with gold nanoparticles led to a near five-fold increase in melanoma cell death when compared to plasma alone^23^. However, the underlying mechanism of this synergic effect is also not well understood. Kong *et al.* introduced a comprehensive and nice review about the interaction of plasma and nanoparticles with cells^24^. It is documented that nanoparticles may preferentially deposit near cancerous cells instead of healthy cells^25^. Therefore, the combination of plasmas and nanoparticles is likely contributed to enhancing selective permeability through the induction of membrane disruption of plasma species leading to facilitated intracellular diffusion of nanoparticles towards diseased sites within a tissue^24^. While there has been some exciting progresses in the field, many challenges still remain with regards to CAP facilitated permeability to include drug encapsulation and high uptake rate.

Therefore, the main objective of this study is to develop a new dual cancer therapeutic method by integrating CAP and novel drug loaded core-shell nanoparticles and evaluate its underlying mechanism for targeted breast cancer treatment. The two-fold approach utilizes CAP exposure as an inducer of cancer cell death with minimal side effects to healthy cells while electrosprayed nanoparticles can contribute to a higher drug encapsulation and sustained delivery. The cytotoxicity and anti-cancer effects of CAP and electrosprayed nanoparticles were evaluated. In addition, the biological mechanism of CAP mediated metastasis on metastatic breast cancer cells (MDA-MB-231) was investigated via quantitative real-time reverse transcription-polymerase chain reaction (qRT-PCR).

## Results

### Physicochemical Properties of Electrosprayed Nanoparticles

**Figure 1:**
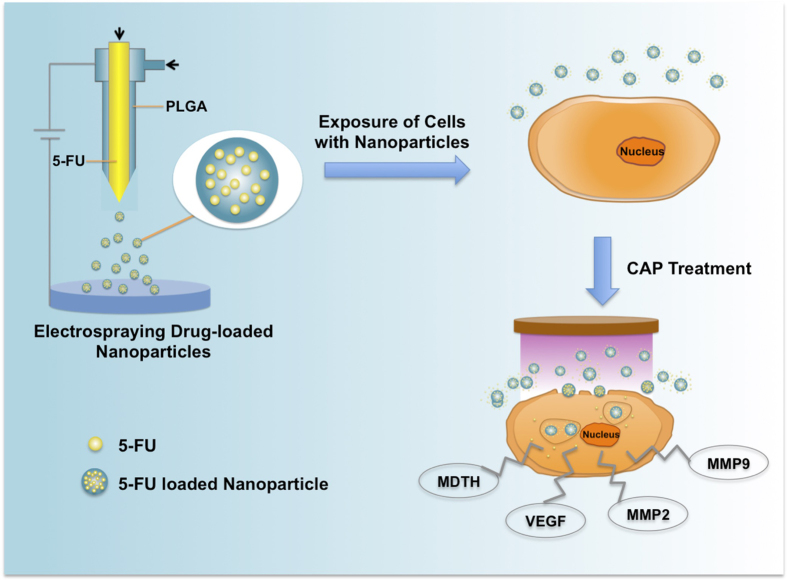
Schematic illustration of electrosprayed core-shell nanoparticle fabrication and CAP facilitated drug delivery.

**Figure 2:**
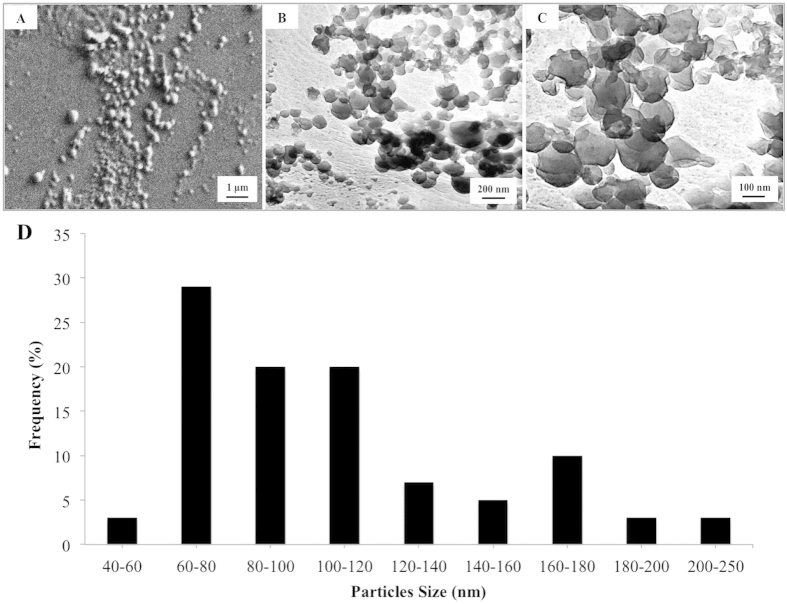
Morphology analysis of drug loaded core-shell PLGA nanoparticles. (**A**) SEM image, and (**B,C**) TEM images of core-shell PLGA nanoparticles with low and high magnifications. (**D**) Nanoparticles size distribution.

Figure 1 summarizes the preparation of 5-FU encapsulated electrosprayed core-shell nanoparticles and integration with CAP for breast cancer treatment. Core-shell nanoparticles were prepared by co-axial electrospraying. Specifically, the core and shell solutions, composed of 1% (w/w) 5-FU in distilled water and 2.5% (w/w) poly(lactic-co-glycolic acid) (PLGA) in acetone, were fed into the inner and outer needles, respectively. Nanoparticle morphology was examined using scanning electron microscopy (SEM) and transmission electron microscopy (TEM). SEM (Fig. 2A) and TEM (Fig. 2B,C) micrographs show individual nanoparticles were produced with uniform spherical morphology and homogenous size distribution with nanoparticle diameters ranging between 60 nm to 120 nm and an average size of 109 nm (Fig. 2D).

**Figure 3:**
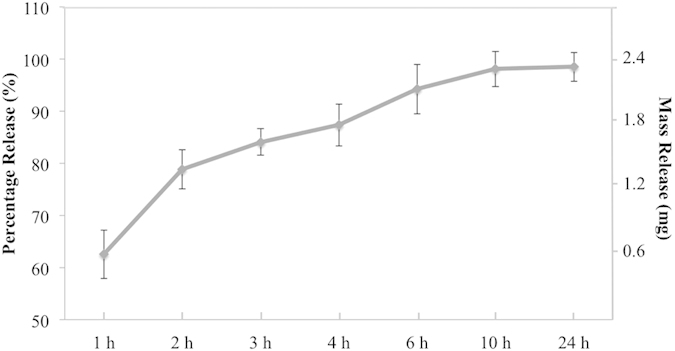
*In vitro* drug release profile of electrosprayed core-shell nanoparticles.

UV absorption analysis revealed nanoparticle drug loading efficiency of 5-FU was 24.1% with accompanying high drug encapsulation efficacy of 64.3%. *In vitro* drug release shows 60% 5-FU release after 1 h followed by sustained release for up to 24 h (Fig. 3).

### *In Vitro* Cytotoxicity of Drug Loaded Nanoparticles

**Figure 4:**
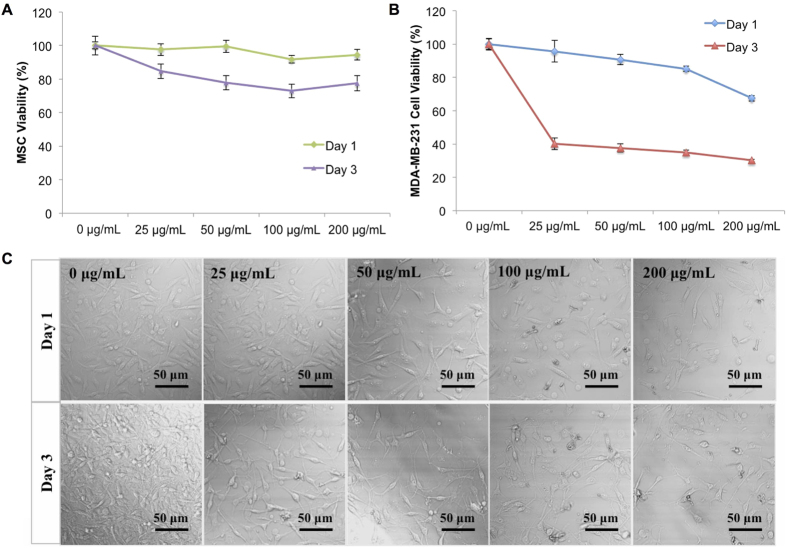
Cytotoxicity of electrosprayed nanoparticles. (**A**) Healthy MSC and (**B**) metastatic MDA-MB-231 cell response to media containing various concentrations of drug loaded nanoparticles after 24 h and 72 h of culture. Data are mean ± standard error of the mean, N = 3. (**C**) Light microscope images of nanoparticles incubated with MDA-MB-231 cells.

Selective cytotoxicity of cancer therapeutics loaded delivery system toward cancer cells not healthy cells is the most desirable feature for various cancer treatments. Therefore, the effects of 5-FU loaded PLGA nanoparticles were evaluated on both healthy cells and breast cancer cells. Human bone marrow mesenchymal stem cells (MSC) were selected as a healthy cell line due to their predominance in bone tissue as well as bone being one of the most popular metastatic sites for breast cancer. Cytotoxicity experiments were carried out for 1 and 3 days with drug loaded nanoparticles exhibiting concentrations ranging from 0 to 200 μg.mL^−1^. After incubation, total cell number was evaluated by CellTiter 96^®^ AQueous Non-Radioactive Cell Proliferation Assay (MTS assay) and viability was calculated based on these results. As shown in Fig. 4A, drug loaded PLGA nanoparticles exhibited limited toxicity against MSCs. No concentration dependent cytotoxic effects of nanoparticles were evident. No adverse effects were noted after 1 day of incubation where greater than 90% MSC viability for all concentrations’ drug loaded nanoparticles treatment was observed. After 3 days, MSC viability remained high with greater than 70% viability even at the highest dose of nanoparticles (200 μg.mL^−1^) suggesting excellent biocompatibility.

**Figure 5:**
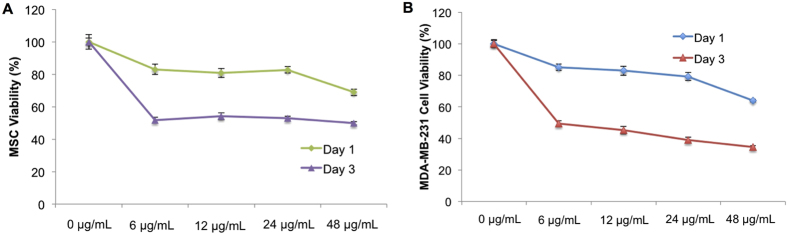
Pure drug exposure to cells. (**A**) Healthy MSCs and **(B**) MDA-MB-231 breast cancer cells were exposed to pure 5-FU at various concentrations for 24 h and 72 h. Data are mean ± standard error of the mean, N = 3.

Metastatic breast cancer cells (MDA-MB-231) were chosen to evaluate the anti-proliferative effects of 5-FU loaded nanoparticles. Results show a concentration and time dependent toxicity of nanoparticles (Fig. 4B). After 3 days of incubation, MDA-MB-231 viability at the maximum concentration (200 μg.mL^−1^) decreased significantly to 34.15% when compared to blank (0 ug.mL^−1^) control. Even at low concentration (25 μg.mL^−1^), nanoparticles exhibited a conspicuous anti-cancer effect. Light microscopy imaging shows MDA-MB-231 morphology exposed to various concentrations of nanoparticles after 1 and 3 days, respectively (Fig. 4C). From this, an apparent dose-dependent response is observed with increasing nanoparticle concentration and administration time which confirms the release and retention of active 5-FU. As comparison, two cell lines (MSC and MDA-MB-231) were also exposed to pure drug with corresponding concentrations (0–48 μg.mL^−1^, determined by the drug loading efficacy). Results showed higher MSC viability was observed at high concentration of pure drug (24 μg/mL, and 48 μg/mL) when both cells were exposed to pure drug (Fig. 5). It indicated breast cancer cells are more sensitive to 5-FU when compared with MSCs which may contribute to the higher viability of MSCs treated with nanoparticles. Some studies also reported the cancer cells are more sensitive to 5-FU compared with MSCs. As illustrated by Kucerova *et al.*, MSCs are more resistant to 5-FU when compared with cancer cells at high drug concentration^26^. In their study, MSC viability is 40% higher than cancer cells at 1000 μg.mL^−1^ 5-FU treatment for 5 days. In contrast, Yu *et al.* found the MDA-MB-231 cell viability is lower than 40% when they are exposed to 130 μg.mL^−1^ 5-FU for 72 h^27^. In addition, it was reported that the nanoparticles preferentially deposit to near cancer cells which might enhance the cytotoxicity response of breast cancer cells^24^.

### Inhibited Metastatic Gene Expression of MDA-MB-231 Cells after CAP Treatment

**Figure 6:**
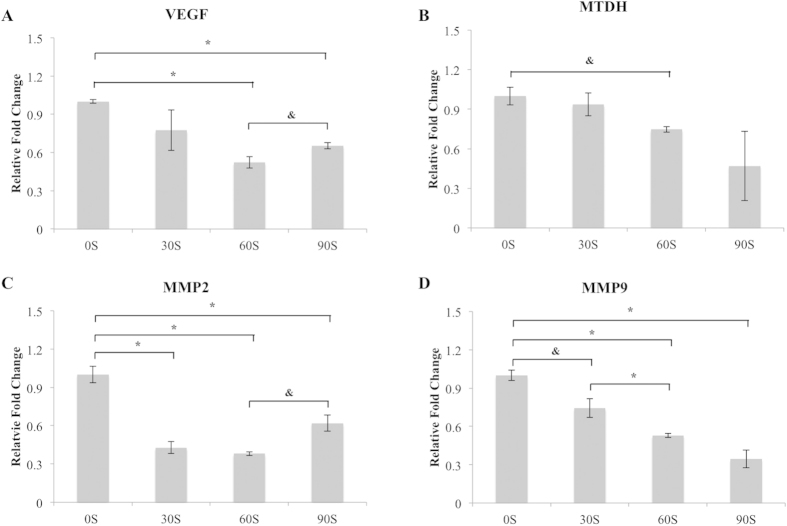
qPCR study. mRNA (VEGF, MMP9, MMP2, MTDH) expression changes during CAP treatment with different time. Bars plotted represent mean and standard error of the mean, n = 3, *p < 0.01, ^&^p < 0.05.

Our previous studies revealed CAP’s selective inhibition of MDA-MB-231 growth with treatment time <90 s^15^. The mechanism governing this observation is still unclear. In this study, qRT-PCR was employed to detect the influence of CAP treatment on metastasis-associated mRNA gene expression. As shown in Fig. 6, CAP treatment significantly decreased metastatic gene expression. Specifically, mRNA expression of VEGF (vascular endothelial growth factor) decreased after 60 s and 90 s CAP treatment when compared to the untreated control. A time dependent down-regulation was observed for MTDH (metadherin) expression as well. In addition, the expressions of genes MMP2 and MMP9 (matrix metalloproteinase-2 and matrix metalloproteinase-9) were also down-regulated after CAP treatment.

### Synergistic Anti-cancer Effect of CAP and Drug Loaded Nanoparticles

**Figure 7:**
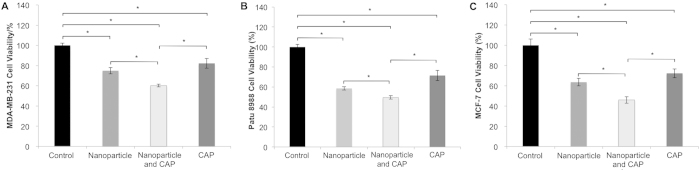
Synergistic effects of CAP and nanoparticles. (**A**) MDA-MB-231, (**B**) PaTu 8988 and (C) MCF-7 cell growth after 24 h culture under various treatment conditions. CAP and drug loaded nanoparticles significantly inhibited cancer cell growth relative to other groups. N = 3, *p < 0.01 when compared to others.

With regards to the advantages of CAP and nanoparticle drug delivery for cancer therapy, we postulate the combination of these two treatments would result in greatly enhanced anti-cancer effects. We quantified MDA-MB-231 breast cancer cell viability via MTS assay. A group in the absence of nanoparticles and without CAP treatment served as a negative control. Single treatment groups including drug load nanoparticles, and CAP only served to evaluate the effects of each treatment independently. Experimental group receiving only CAP was treated for 60 s. All groups were incubated 24 h after initial treatments. As shown in Fig. 7A, all groups presented inhibited breast cancer cell proliferation when compared to untreated control. The group receiving both CAP and nanoparticle treatment exhibited greater anti-proliferative effects on breast cancer cells indicating beneficial combinatorial anticancer efficacy. Specifically, MDA-MB-231 cell numbers in group that treated by both CAP and nanoparticles decreased 39.8% when compared to control after 1 day culture. When PaTu 8988 and MCF-7 cells were treated with CAP and nanoparticles, the cell viability decreased to 49.5% and 46.1%, respectively, when compared to untreated controls (Fig. 7B,C).

### Enhanced cellular internalization of nanoparticles by CAP treatment

**Figure 8:**
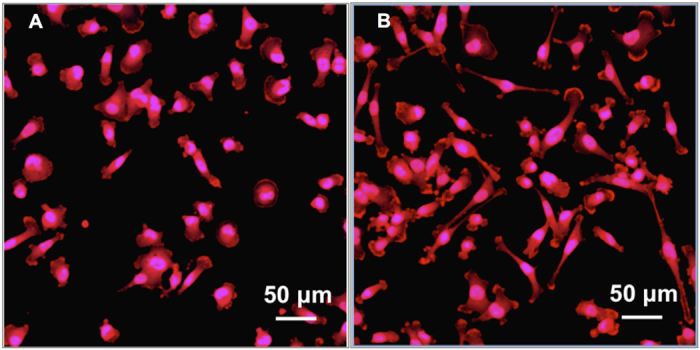
Cell morphology after CAP treatment. Confocal images of MDA-MB-231 cells with (**A**) CAP exposure compared to (**B**) untreated control. Cells were fixed and stained after 60 s CAP exposure and incubated 1 h.

**Figure 9:**
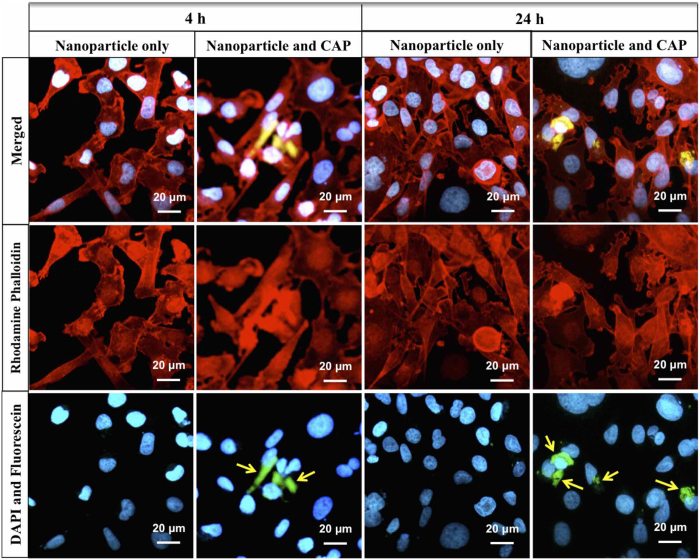
Enhanced cellular uptake of nanoparticles by CAP. Confocal micrographs of MDA-MB-231 cells treated with CAP and nanoparticles after 4 h and 24 h incubation. Arrows indicate the uptake of nanoparticles. Improved nanoparticle uptake was evident after CAP treatment.

In order to detect the influence of CAP treatment on cell morphology, MDA-MB-231 cells were stained and imaged. Confocal microscope images illustrated apparent morphology change after CAP treatment (Fig. 8). The microvilli and pseudopodia of cells were diminished by the CAP. Then, cellular uptake of nanoparticles was evaluated by laser scanning confocal microscopy. MDA-MB-231 cells were double stained with rhodamine phalloidin (red) and 4′,6-Diamidine-2′-phenylindole dihydrochloride (DAPI, blue) for actin and nucleus characterization, respectively. PLGA nanoparticles were conjugated with fluorescein (green) for tracing. Fluorescence imaging of CAP-assisted nanoparticle internalization illustrates nanoparticle localization around the cell nucleus (Fig. 9) after 1 h of CAP treatment. In contrast, no visible fluorescence signal was detected on cells incubated with nanoparticles alone indicating no cellular uptake of nanoparticles. Even after 24 h incubation still no nanoparticles internalization was observed amongst non-CAP treated samples.

## Discussion

Electrospraying is an effective and efficient tool to fabricate core-shell nanoparticles. During the electrospraying process, the polymer solution dispensing from the nozzle readily forms core-shell droplets of several nanometers in diameter under an increasing electric field. The benefits of co-axial electrospraying include: a) ability to separate organic and aqueous phases and thus incorporate drugs with no exposure to harmful organic solvents; b) ability to produce small and uniform particle size; c) absence of particle aggregation and coagulation; d) ease of control of the operating parameters; e) scalability^28^,^29^. Studies have shown that particle size significantly influences the duration of sustained drug release which can be attributed to the inverse relationship between surface areas to volume. Generally, a larger surface area to volume ratio contributes to increased drug release rates^30^. PLGA, the polymeric shell material used in this study, is the most characterized biodegradable material for use as a drug carrier for controlled release with suitable tensile strength and degradation rate ensuring structural integrity of particles during the delivery process^31^. Studies have illustrated the capacity of electrospraying technique in fabricating PLGA particles with clear core-shell structure^32^. *In vitro* studies have shown biphasic degradation of PLGA nano/microparticles with a rapid initial degradation followed by a much slower degradation phase (20–30 days)^33^. PLGA nanoparticles have been widely studied owing to their excellent biocompatibility and cytotoxicity towards various cell lines and have been approved by FDA and European Medicine Agency for many drug delivery applications^34^. Mura *et al.* investigated the lung toxicity of PLGA nanoparticles displaying various surface chemistry and surface charge on human bronchial Calu-3 cells^35^. PLGA nanoparticles were tuned by coating with chitosan, Poloxamer, and poly (vinyl alcohol) for grafting positive and negative as well as neutral charges. They found the cytotoxicity of PLGA nanoparticles was negligible with no elicitation of an inflammatory response. Morphological examination of our electrosprayed core-shell PLGA nanoparticles revealed the formation of particles with uniform size. In addition, drug loading characterization showed high drug loading efficacy. More importantly, 5-FU loaded nanoparticles presented low cytotoxicity to healthy human cells (MSCs) but high breast cancer cell anti-proliferative effects indicating they are well-suited for use as an anti-cancer drug delivery device.

VEGF is a protein which stimulates the growth and formation of the circulatory systems and blood vessels (vasculogenesis and angiogenesis). With regards to its role in cancer, VEGF facilitates the presence of nutrients and oxygen to cancer cells. More blood supply promotes cancer cell proliferation and inhibits cell apoptosis. Therefore, down-regulation of VEGF mRNA expression can serve as an indicator of the inhibited cancer progression. A more direct indicator of cancer progression, MTDH is an oncogene which promotes breast cancer cell proliferation. MTDH is typically overexpressed in greater than 40% of breast cancers. In addition, MTDH is attributed to the development of chemoresistance as well as increasing metastatic potential^36^. MMP2 and MMP9 share similar biological function where they play a key role in the breakdown of extracellular matrix. During cancer development, MMP2 and MMP9 degrade the basement membrane as well as promote tumor cell metastasis to distant tissues and/or organs. In our study, for the first time, we found CAP treatment down-regulated all four markers. Specifically, 60 s CAP treatment had comparable effects with 90 s treatment for MDA-MB-231 cells. Based on these results, CAP treatment might reduce drug resistance during chemotherapy as well as control breast cancer metastasis to other tissues or organs through down-regulation of metastasis-related gene expression.

Although the underlying mechanism of CAP mediated cell behavior is not totally clear, mounting evidence continues to show this process is highly related to intracellular reactive oxygen and nitrogen species. Kalghatgi *et al.* found a dose-dependent response of CAP on proliferation and apoptosis of mammalian breast epithelial cells^18^. They found the reactive oxygen species generated by CAP interacts with intracellular organic components within the cells to extend the availability of these species for chemical modulation. Low levels of reactive oxygen species are known to promote cell proliferation, while greater concentrations of intracellular reactive oxygen species result in replication arrest or single-stranded DNA breaks formation. With regards to cancer therapy, clinical applications will be concentrated on controlling the dosage of CAP suitable for solid tumor ablation with minimal normal cell disruption. Another biomolecule presented in CAP which has large implications in cancer therapy is NO. NO is well known to be a powerful agent to sensitize chemotherapeutic drugs for long-term activity against a variety of cancers^37^. Therefore, the current treatment strategy by combining CAP and conventional chemotherapeutic agents could further minimize drug resistance in cancerous cells. Our qRT-PCR results confirmed the down-regulation of chemoresistance-associated gene expression as a function of CAP treatment time.

In this study, we observed a synergistic interaction of CAP and nanoparticles on cancer remediation. Amongst some potential hypotheses, this synergy may be related to the induction of a porous nanostructure resulting in more reactive plasma species trapping^24^. The trapped reactive species could extend their half-life and ensure safe delivery to the target area so as to cancer area because nanoparticles prefer to deposit near cancer cells^25^. Therefore, nanoparticles could become a carrier of plasma produced ROS/RNS to effectively deliver the reactive plasma species deep into the diseased tissue. In addition, energy deposition caused by plasma and/or nanoparticles might induce temporal opening of channels and pores in a tissue and eventually lead to enhanced interaction between plasma/nanoparticles and tissues^24^. In our studies, breast cancer cells were seeded in culture medium containing nanoparticles. CAP was then applied directly to the cell culture in order to maximize the interaction between plasma and nanoparticles. Cell proliferation results (Fig. 7) confirmed that the group treated with CAP and nanoparticles improved anticancer efficacy relative to groups treated with nanoparticles or CAP treatment only.

Clinical evidence suggests the efficacy of nanoparticle therapeutics is highly dependent on active cellular uptake^5^,^38^. The uptake of polymeric particles is influenced by particle size, shape, surface properties, and concentration. Chemical surface modification techniques have been widely employed to enhance the interaction between cell and polymeric particles. However, the use of harsh organic solvents is a major concern during these processes which could impose problems toward cell viability^39^. Consequently, solvent-free technologies such as the CAP technique used here has gradually become the subject of intense research for facilitated cellular uptake of nanoparticles. Due to the complex nature of CAP composition, a greater understanding of its effects to the integrity of the cell membrane of breast cancer cells leading to enhanced nanoparticles internalization is warranted. Yan *et al.* found out that plasma treated silica nano particles exhibited longer endurance and higher dielectric breakdown strength under the constant electric stress^40^. The study further implied that this method could be beneficial to any organic nanocomposite by improving mechanical strength, thermal stability and electrical properties. In addition, some studies have investigated plasma modification of PLGA. For example, Hasirci *et al.* observed an increased hydrophilicity when PLGA films were treated with plasma^41^ which may be due to the incorporation of nitrogen and oxygen functional groups upon the material’s surface. A more hydrophilic surface readily leads to more specific protein adsorption and greater cell adhesion^42^. These physical and chemical improvements of nanoparticles may partly contribute to better cellular internalization of nanoparticles when compared to untreated nanoparticles. In addition, the alteration of architecture (Fig. 8) and functionality of cancer cells might lead to improved cellular uptake of nanoparticles. In our previous study, atomic force microscope detection also showed the cell shape and morphology change with CAP treatment^43^. Theses changes are regarded associating with a wide array of cellular functions including absorption, secretion, and mechanotransduction.

Therefore, in our study, we are seeking a new cancer therapy by integrating CAP and nano drug delivery system. The synergistic inhibition for cancer cells refers to two aspects. Firstly, the combination of CAP and drug delivery system is expected to reduce the drug resistance of breast cancer cells for enhanced long-term effective chemotherapy. Targeting MTDH could be an effective strategy to enhance chemotherapy efficacy. Therefore, one synergistic effect of the combined therapy is that CAP reduced the drug resistance of cancer cells which in turn enhance the efficacy of anti-cancer agent. Secondly, we also found the CAP treatment improved the endocytosis. This is another synergistic effect of CAP and drug loaded nanoparticles therapy.

## Conclusions

Electrosprayed nanoparticles exhibiting homogenous size distribution and high encapsulation efficacy of anti-cancer agent have been developed here to serve as a sustained delivery system. Cell studies illustrate the effectiveness of combining drug loaded nanoparticles and CAP as a novel dual cancer treatment for the synergetic inhibition of cancer cell growth when compared to each single treatment. In addition, CAP treatment down-regulated metastasis related gene expression (VEGF, MMP9, MMP2, MTDH) which can play a critical role in resolving drug resistance. Thus, the combination of CAP and drug delivery may lead to a shift in the paradigm of cancer therapy.

## Methods

### Electrosprayed Nanoparticle Synthesis and Characterization

Nanoparticles were fabricated via a custom core-shell needle composed of a 20 G outer and 26 G inner diameter needle connected to a high voltage power supply. PLGA (50:50 with inherent viscosity range from 0.55 to 0.75 dL/g in Hexafluoroisopropanol, Sigma-Aldrich) was dissolved in acetone at a concentration of 2.5% w/v and delivered through the shell feed inlet. 5-FU was re-suspended in water at a concentration of 10 mg/mL and pumped through the core feed inlet. A glass petri dish containing 10 mL water was placed beneath the needle as a collector. The electrosprayed nanoparticle solution was stirred two hours in air to allow all the solvent evaporation prior to freezing and lyophilization.

SEM (Zeiss NVision 40 FIB) and TEM (JEOL 1200 EX) were used to characterize nanoparticle morphology and size homogeneity. Prior to imaging, all samples were sputter-coated with gold to prevent electron beam damage. Size distribution of nanoparticles was determined by imageJ (National Institutes of Health, USA) based on collected micrographs.

### Drug Release Profile

*In vitro* 5-FU release was performed at 37 °C. Briefly, 10 mg 5-FU encapsulated nanoparticles were dispersed in 1.5 mL PBS solution in a micro-centrifuge tube. At predetermined time points, 1, 2, 3, 4, 6, 10, and 24 h, samples were centrifuged at 10000 rpm for 6 min. A 100 μL fraction of supernatant was collected and replaced with fresh PBS. The absorbance of supernatant was read by a Thermo Scientific Multiskan GO Spectrophotometer at 265 nm wavelength light. All samples were prepared in quintuplicate. 5-FU encapsulation efficacy and loading efficacy of nanoparticles were determined as follows:


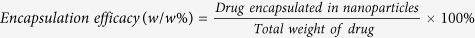



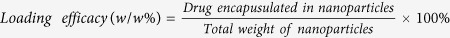


### Cell Culture and Toxicity Studies

MSC and MDA-MB-231 cell lines were studied for biocompatibility and cytotoxicity of nanoparticles. Primary human bone marrow MSCs were harvested from healthy consenting donors in Texas A&M Health Science Center, Institute for Regenerative Medicine. MSCs were cultured in alpha minimum essential medium supplemented with 16.5% fetal bovine serum, 1% (v/v) L-glutamine, and 1% penicillin/streptomycin solution under a condition of 37 °C and 5% CO_2_/95% air environment. MDA-MB-231 cells media consisted of Dulbecco’s modified eagle medium supplemented with 10% fetal bovine serum and 1% penicillin/streptomycin. Prior to each test, cells were seeded at a density of 5,000 cells/well in 96-well plates. Both cell lines were incubated with varying concentrations of nanoparticles in a range of 0–200 μg.mL^−1^. MTS cell viability assay was performed after incubation of cells with drug encapsulated nanoparticles for 24 and 72 hours. The absorbance measurement was taken at 490 nm. The viability of cells cultured in normal media (without nanoparticles) was used to normalize the experimental groups (with different concentrations nanoparticles). In addition, MSC and MDA-MB 231 cells were treated with pure drug. At the same time points, the cell number was counted by MTS assay and cell viability was calculated. The concentration of pure drug was determined by the nanoparticle amount used in above experiment and the acquired drug loading efficacy.

### mRNA qRT-PCR

Four metastasis-associated messenger RNA (mRNA) gene expression (VEGF, MMP9, MMP2, MTDH) was detected regarding different times of CAP treatment. Briefly, MDA-MB-231 cells were seeded in 96-well plate and incubated 24 h. CAP treatment homogeneity was then conducted with various times and cells were allowed growth for another 24 h. Total RNA was isolated from the cell clones using Trizol reagent (Invitrogen) per manufacturer’s instruction. The NanoDrop1000 Spectrophotometer (ThermoScientific) was employed to quantify the amount of RNA samples. Specifically, 200 ng of total RNA was utilized for reverse transcription using the iScript cDNA Synthesis Kit (Bio-Rad). qRT-PCR was performed using the ABI 7300 Real-Time PCR System (Applied Biosystems) to verify gene expression in various cell clones. A final volume of 20 μL for each reaction included 0.5 μL (20 μM) of each primer (IDT, Coralville, IA, USA), 2 μL of 10-fold diluted cDNA, 10 μL SYBR Green PCR Master Mix (Applied Biosystems) and 6 μL nuclease-free water. The conditions for qRT-PCR were kept at 50 °C for 2 minutes, 95 °C for 10 minutes, followed by 40 cycles of 95 °C for 15 seconds and 60 °C for 60 seconds. Dissociation curves were created for each primer set to confirm the specificity of amplifications. 18 S ribosome RNA was used to normalize the Mean Quantity values of target genes mRNAs expression. The primer sequences are for MTDH: forward, 5′-CAAACCAAATGGGCGGACTG-3′ and reverse, 5′-GTCAATCTCTGGTGGCTGCT-3′; for MMP2: forward, 5′-TGGACCCAGAGACAGTGGAT-3′ and reverse, 5′-TTCGAGAAAACCGCAGTGGG-3′; for MMP9: forward, 5′-CAGTCCACCCTTGTGCTCTT-3′ and reverse, 5′-CCCGAGTGTAACCATAGCGG-3′; for VEGFA: forward, 5′-AAGAAATCCCGTCCCTGTGG-3′ and reverse, 5′-GCAACGCGAGTCTGTGTTTT-3′; for 18 S: forward, 5′-GCCGCTAGAGGTGAAATTCTTG-3′ and reverse, 5′-CATT CTTGGCAAATGCTTTCG-3′. The cells without CAP treatment is selected as control.

### Anticancer Effects of Combined CAP and 5-FU Encapsulated Nanoparticles

MBA-MD-231 cells were seeded in 96-well plates at a density of 5,000 cells/well. After 24 h culture, various conditions were used to treat cells. Experimental groups with 200 μg.mL^−1^ nanoparticle containing media were exposed to 60 s CAP treatment. Control groups included cells with normal media (as 100% viability to normalize others), normal media with nanoparticles only, and normal media with 60 s CAP treatment only. The cell viability was evaluated after treatments for 24 h using MTS assay. In addition, a pancreatic cancer cell line (PaTu 8988), and another breast cancer cell line (MCF-7) were used to validate the efficacy of the combined therapy.

### Cell Imaging

Cell morphology change after CAP treatment was investigated by a laser scanning confocal microscope (LSCM 710, Zeiss). MDA-MB-231 cells were fixed after 1 h of treatment and double-stained with rhodamine phalloidin (Life technologies) and DAPI (Sigma-Aldrich). To determine nanoparticle cellular uptake, nanoparticles were fluorescently labeled with fluorescein. Briefly, 0.25 mg fluorescein (Sigma-Aldrich) and 50 mg PLGA were dissolved in 2000 mg acetone and used to fabricate electrosprayed nanoparticles as previously described. 10,000 cells were placed on 35 mm diameter petri dish with a 14 mm circular glass microwell for 24 h prior to treating with nanoparticle medium and CAP. The drug encapsulated nanoparticles were diluted with culture media to 200 μg.mL^−1^. CAP treatment was focused on the microwell area. After 4 h and 24 h incubation with nanoparticles and CAP treatment, cells were fixed with 10% formalin for 15 min followed double-staining with rhodamine phalloidin and DAPI.

## Additional Information

**How to cite this article**: Zhu, W. *et al.* Synergistic Effect of Cold Atmospheric Plasma and Drug Loaded Core-shell Nanoparticles on Inhibiting Breast Cancer Cell Growth. *Sci. Rep.* **6**, 21974; doi: 10.1038/srep21974 (2016).
